# Energy-Dense and Low-Fiber Dietary Pattern May Be a Key Contributor to the Rising Obesity Rates in Brazil

**DOI:** 10.3390/ijerph21081038

**Published:** 2024-08-07

**Authors:** Iuna Arruda Alves, Mahsa Jessri, Luana Silva Monteiro, Luiz Eduardo da Silva Gomes, Taís de Souza Lopes, Edna Massae Yokoo, Rosely Sichieri, Rosangela Alves Pereira

**Affiliations:** 1 Graduate Program in Nutrition, Federal University of Rio de Janeiro (UFRJ), Rio de Janeiro 21941-590, RJ, Brazil; 2 Food, Nutrition and Health Program, Faculty of Land and Food Systems, The University of British Columbia, Vancouver, BC V6T 1Z4, Canada; mahsa.jessri@ubc.ca; 3 Centre for Health Services and Policy Research (CHSPR), Faculty of Medicine, The University of British Columbia, Vancouver, BC V6T 1Z3, Canada; 4 Institute of Food and Nutrition, Federal University of Rio de Janeiro (UFRJ), Macaé 27930-560, RJ, Brazil; luananutrir@gmail.com; 5 Graduate Program in Statistics, Institute of Mathematics, Federal University of Rio de Janeiro (UFRJ), Rio de Janeiro 21941-909, RJ, Brazil; luizeduardo@dme.ufrj.br; 6 Department of Quantitative Methods, Center of Exact Sciences and Technology, Federal University of the State of Rio de Janeiro (UNIRIO), Rio de Janeiro 22290-240, RJ, Brazil; 7 Department of Social and Applied Nutrition, Federal University of Rio de Janeiro (UFRJ), Rio de Janeiro 21941-590, RJ, Brazil; taislopes@nutricao.ufrj.br (T.d.S.L.); roapereira@gmail.com (R.A.P.); 8 Department of Epidemiology and Biostatistics, Institute of Collective Health, Fluminense Federal University (UFF), Niterói 24030-210, RJ, Brazil; eyokoo@gmail.com; 9 Institute of Social Medicine, Rio de Janeiro State University (UERJ), Rio de Janeiro 20550-013, RJ, Brazil; rosely.sichieri@gmail.com

**Keywords:** obesity, nutrition surveys, diet, feeding behavior, adult

## Abstract

Hybrid methods are a suitable option for extracting dietary patterns associated with health outcomes. This study aimed to identify the dietary patterns of Brazilian adults (20–59 years old; *n* = 28,153) related to dietary components associated with the risk of obesity. Data from the 2017–2018 Brazilian National Dietary Survey were analyzed. Food consumption was obtained through 24 h recall. Dietary patterns were extracted using partial least squares regression, using energy density (ED), percentage of total fat (%TF), and fiber density (FD) as response variables. In addition, 32 food groups were established as predictor variables in the model. The first dietary pattern, named as energy-dense and low-fiber (ED-LF), included with the positive factor loadings solid fats, breads, added-sugar beverages, fast foods, sauces, pasta, and cheeses, and negative factor loadings rice, beans, vegetables, water, and fruits (≥|0.15|). Higher adherence to the ED-LF dietary pattern was observed for individuals >40 years old from urban areas, in the highest income level, who were not on a diet, reported away-from-home food consumption, and having ≥1 snack/day. The dietary pattern characterized by a low intake of fruits, vegetables, and staple foods and a high intake of fast foods and sugar-sweetened beverages may contribute to the obesity scenario in Brazil.

## 1. Introduction

Obesity is recognized as a global epidemic and is a public health concern due to its association with an increased risk of non-communicable diseases [1,2]. In Brazil, the prevalence of obesity among adults increased from 18.5% to 26.8% between 2006 and 2019 [3,4]. Moreover, in 2023, in Brazilian state capitals, among the adults, the prevalence of overweight was 61.4% and obesity was 24.3% [5]. According to Estivaleti et al. [3], the trend towards an increase in the prevalence of obesity will likely continue and the projections indicate that, by 2030, 30% of the Brazilian adult population will be affected by obesity.

An unhealthy diet is a major risk factor associated with obesity, and dietary pattern analysis is considered an appropriate analytical strategy to ascertain the association between diet and health outcomes [6]. This approach takes into account the complexity of diet, rather than solely focusing on specific foods or nutrients [6,7,8]. Among the methods available to identifying dietary patterns, hybrid methods that combine hypothesis-oriented approaches and statistics procedures allow for the extraction of dietary patterns that may predict the outcome of interest [9]. One of these methods is partial least squares (PLS) regression, which maximizes the explanation of the variability in food and nutrient intake or biomarkers associated with the outcome [9]. Dietary pattern analysis has been applied in national [10] and international [11,12] studies that investigated the association between dietary patterns and obesity.

In Brazil, a cross-sectional study carried out with adult residents in a low-income urban area found that a Western eating pattern, composed of fast foods, added-sugar beverages, and sweets, was directly associated with body mass index (BMI) and waist circumference among women. These associations remained after adjustment for age, education, smoking, physical activity, and energy intake. Moreover, a traditional pattern, based mainly on rice and beans, exhibited an inverse association [10]. In a cohort study with young Brazilian adults (23–25 years old), the Bar eating pattern, composed of alcoholic beverages, processed meats, and snacks, was associated with a greater prevalence of excess weight and abdominal obesity for both sexes [13]. Muniz et al. [14] analyzed data from the same cohort and found that unhealthy eating patterns, characterized by fast foods, processed meats, sauces, and alcoholic and added-sugar beverages, were associated with a higher risk of obesity and excess body fat percentage, independent of sex, skin color, family income, schooling, smoking, physical activity, and reporting quality.

The identification of dietary patterns, especially when based on hypothesis-driven methods, may provide important information to support health promotion actions. Therefore, this study aimed to identify the dietary patterns related to dietary components that are potentially associated with excessive weight gain.

## 2. Materials and Methods

### 2.1. Ethical Statement

In the 2017–2018 Brazilian National Dietary Survey (NDS), the study was deemed exempt by the Committee of Ethics in Research of the Institute of Social Medicine, State University of Rio de Janeiro (review number 4.316.087), under the Brazilian National Health Council Resolution number 466/2012 and Operational Act number 001/2013, since the data were de-identified and are publicly available (www.ibge.gov.br (accessed on 15 September 2022)).

### 2.2. Study Design and Population

This cross-sectional study analyzed the data from the Brazilian National Dietary Survey (NDS) conducted by the Brazilian Institute of Geography and Statistics (acronym in Portuguese: IBGE) from the 2017–2018 Household Budget Survey (HBS). The HBS adopted a cluster sample design with two stages of selection. The NDS was carried out in a randomly selected subsample of the households included in the HBS, obtaining data on individual food consumption of subjects aged 10 years old and over. In 2017–2018, the HBS main sample included 57,920 households, and the NDS subsample encompassed 20,112 households [15]. Data from 28,153 adults (20 to 59 years old) were analyzed after excluding pregnant and lactating women (*n* = 1200).

### 2.3. Dietary Data Collection

Two 24 h recalls (24hR) were applied on non-consecutive days within a one-week span by means of in-person interviews using procedures based on the Multiple-Pass Method [16] and a tablet-supported software including a database with 1832 food items. The individuals reported all foods and beverages (including water) consumed during the day before the interview, reporting detailed information on portion size, cooking method, time and location of consumption, and named the meal or consumption occasion (breakfast, lunch, dinner or snack) [15]. For specific foods, the possible addition of 12 items was questioned using yes/no questions: olive oil, margarine/butter, sugar, non-caloric sweetener, honey, molasses, mayonnaise, ketchup, mustard, soy sauce, grated cheese, and whipping cream [15].

Energy and nutrient intake were estimated using the Brazilian Food Composition Table (TBCA) v. 7.0 [17], taking into account items (see above) that may have been added to specific foods. To this end, standardized procedures were considered since no information on the amount added to food was available. A maximum of 20% of the amount consumed (in grams) was estimated, summing up the addition of all fat-based items (for example, if the participant added olive oil and grated cheese, the intake of each one was estimated as 10% of the amount reported). A maximum of 10% of the amount consumed was estimated for the total addition of sugar, honey, molasses, ketchup, mustard, and soy sauce.

The foods reported in the first 24 h recall were categorized into 32 food groups according to their nutritional characteristics: coffee and tea; water; rice; beans; sugars; beef and pork; bread; vegetables; solid fats; poultry; fruit juice; roots and tubers; fruits; fast food; cookies and crackers; pasta; sugar-sweetened beverages; vegetable oils; eggs; cakes and baked goods; candies, sweets, and desserts; processed meats; cheeses; corn and corn dishes; milk; whole grains; dairy beverages; fish; non-caloric sweeteners; soups and broths; sauces; alcoholic beverages (Supplemental Table S1).

### 2.4. Dietary Pattern Analysis

The partial least squares (PLS) regression method was applied to identify dietary patterns, which used the following response variables: energy density (ED) (kcal/gram), proportion (%) of energy intake provided by total fat (%TF), and fiber density (FD) (gram/1000 kcal). The response variables were chosen considering the World Health Organization recommendations [18,19] and studies pointing out that these dietary components are related to an increased risk of obesity [11,20]. The energy density was calculated as the ratio between energy intake and the amount consumed (kcal/gram), excluding beverages (Equation (1)). Fiber density was estimated by calculating the grams of fiber intake per 1000 kcal (Equation (2)), and %TF represented the proportion of total daily energy intake provided by total fat (Equation (3)). The predictor variables, the intake (in grams) of the 32 food groups, were standardized.

(1) $$Energy\;density=\frac{Total\;energy\;intake\;(kcal)}{Amount\;of\;solid\;foods\;consumed\;(gram)}$$

(2) $$Fiber\;density=\frac{Total\;fiber\;intake\;\left(gram\right)}{Total\;energy\;intake\;(kcal)}\times 1000$$

(3) $$\%\;Total\;fat=\frac{Energy\;from\;total\;fat\;\left(kcal\right)}{Total\;energy\;intake\;\left(kcal\right)}\times 100$$

Food groups with a factor loading ≥|0.15| were retained in the patterns. The standardized individual dietary pattern scores were categorized into quintiles. The PLS analysis was performed using the “PROC PLS” command in SAS OnDemand for Academics.

### 2.5. Characterization of the Study Population

The studied population was characterized according to the following sociodemographic variables: (a) sex (female or male); (b) age group (20 to 39 years old and 40 to 59 years old); (c) urban or rural household situation; (d) monthly per capita family income (estimated from the sum of household incomes divided by the number of household members and classified according to multiples of Brazil official minimum wage [MW] at the middle of the study, i.e., USD 298.50 in January 2018: <0.5, 0.5–1.0, 1.0–2.0 and >2 MW); (e) being on a diet or not at the time of the interview; (f) away-from-home (if at least one food was eaten away from home throughout the day) or at-home only food consumption; and (g) snacking habits (no snacks or at least one snack reported throughout the day) [15].

### 2.6. Statistical Analysis

The Pearson’s correlation coefficient between the predictor factor loadings and response variables was calculated, and the scores of the dietary pattern were identified. Dietary pattern scores were categorized into quintiles. Means and proportions (%) of socio-demographic and dietary habit variables were estimated for the total population and according to dietary pattern quintiles.

All analyses considered the complex sample and study design effect and were performed using SAS On Demand (welcome.oda.sas.com (accessed on 20 April 2024)). Differences in means and proportions across the analyzed categories were evaluated based on whether the 95% confidence intervals (95%CIs) overlapped.

## 3. Results

**Figure 1:**
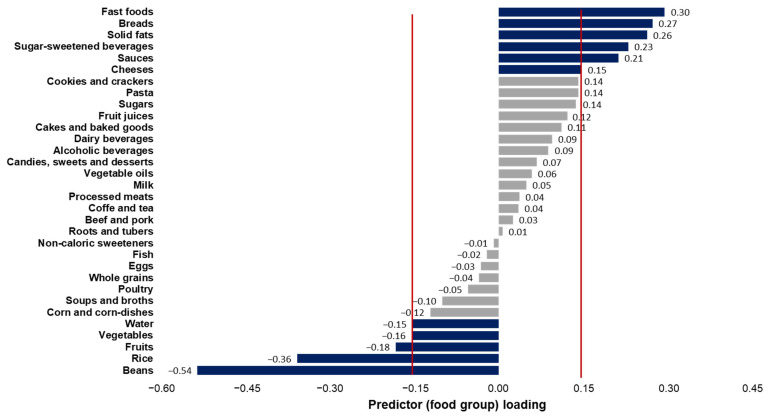
Factor loadings of food groups included the “energy-dense and low-fiber” adults’ dietary pattern extracted using partial least squares. Brazil, National Dietary Survey, 2017–2018. Note: Food groups with factor loadings ≥|0.15| were retained in the dietary pattern.

Three dietary patterns, which together, explained 48.8% of the variation in the response variables, were extracted. The first dietary pattern explained 4.2% of the food consumption variation and most of the variation in the response variables (32.0%, with ED accounting for 46.7%, %TF 2.5%, and FD 46.7%). Patterns 2 and 3 explained 3.8% and 4.3% of the variability in food consumption by, respectively. Regarding response variable variability, these patterns explained 12.0% and 4.8%, respectively. The food groups retained in patterns 2 and 3 did not present an interpretable composition. Thus, only the first pattern was considered in the analysis due to its interpretability. In the first dietary pattern, the retained foods with positive factor loadings were fast foods, breads, solid fats, sugar-sweetened beverages, sauces, and cheeses. Additionally, beans, rice, fruits, vegetables, and water were retained with negative factor loadings (Figure 1). Therefore, this pattern was named the energy-dense and low-fiber pattern (ED-LF).

The ED-LF pattern scores were directly correlated with ED (r = 0.686) and inversely correlated with FD (r = −0.699), while a weak positive correlation was observed with %TF (r = 0.156). The highest correlation coefficient between the predictors (food groups) and the ED-LD dietary pattern scores was for fast foods (r = 0.361), whereas the lowest negative correlation coefficients were estimated for beans (r = −0.639) and rice (r = −0.431). The highest positive correlation coefficients between energy density and food groups were estimated for breads (r = 0.249) and fast foods (r = 0.243); the lowest negative coefficients were estimated for beans (r = −0.327) and fruits (r = −0.269), while for FD and %TF, the highest coefficients were estimated for beans and vegetables (r = 0.673 and r = 0.226, respectively). All coefficients were significant with *p* < 0.01 (Table 1).

Overall, the mean ED-LF dietary pattern score was 0.10 and the quintile score means varied from −1.53 (quintile 1: least adherence; healthier) to 1.54 (quintile 5: most adherence; unhealthier). In general, the mean energy density was 1.78 kcal/gram, mean fiber density was 13.2 g/1000 kcal, and mean total fat intake was 29.8% of the total energy intake. Across the quintiles of the ED-LF dietary pattern scores, the energy density varied between 1.35 and 2.27 kcal/gram, the fiber density ranged between 22.0 and 7.5 g/1000 kcal, and the total fat intake ranged between 27.7% and 30.9% of the total energy intake. For all food groups positively loaded in the ED-LF dietary pattern, the mean intake increased across the quintiles of the dietary pattern scores, and the greatest increments were observed for sauces, fast foods, and sugar-sweetened beverages. The mean intake of sauces was 0.3 g/day in quintile 1 and 5.7 g/day in quintile 5; for fast foods, the mean daily intake varied between 12.5 g and 97.3 g, and for sugar-sweetened beverages, it was between 34.7 and 203.5 g. For all inversely loaded food groups, the mean daily intake of the food groups was reduced across the quintiles of the ED-LF food groups. The greatest reductions were observed for beans and fruits. The mean bean intake in quintile 1 was 415.1 g/day and in quintile 5, it was 76.3 g/day. The mean fruit intake was 113.7 g/day in quintile 1 and 30.9 g/day in quintile 5 (Table 2).

Most of the adults lived in the Southeast region (42.8%) and in urban areas (86.3%), and 31.3% earned between 1 and 2 minimum wage equivalents. Women comprised 50.2% of the investigated population and 54.6% of examined individuals were between 20 and 40 years old. Regarding dietary habits, 13.1% reported being on a diet, 16.9% reported the use of supplements in the 30 days prior to the interview, 47.9% reported away-from-home eating, and 85.7% reported having at least one snack per day (Table 3).

Quintile 5 of the ED-LF dietary pattern scores included 20.8% of the women and 25.0% of the men, and 19.7% of the 20–40-year-old individuals and 25.5% of those >40 years old. In urban areas, 24.4% of the individuals were classified in quintile 5 while in rural areas, this proportion was 13.1%. In the lowest income level, 14.9% of the adults were classified into quintile 5 and in the highest income level, 28.0% were classified into quintile 5. Among the individuals who reported being on a diet, 14.4% were categorized into quintile 5, while among those who were not on a diet, this proportion was 24.1%. Comparing the individuals that reported using or not using supplements, there was no difference in the distribution according to the quintiles of the ED-LF dietary pattern (quintile 5: 24.6% and 22.5%). Among those who reported away-from-home food consumption, 26.2% were classified into quintile 5, while among the individuals who reported only at-home eating, this proportion was 19.8%. Comparing individuals who had at least one snack and those reporting not snacking, the proportion of individuals in quintile 5 was 23.5% and 18.8%, respectively (Table 3).

Quintile 1 of the ED-LF dietary pattern scores included 22.4% of the men and 14.1% of the women and 19.6% of the individuals aged between 20 and 40 years old and 17.1% of those over 40 years old. In rural areas, 31.1% of these individuals were classified into quintile 1 while in urban areas, this proportion was 16.2%. In the lowest income level, 23.6% of the adults were classified into quintile 1, while in the highest income level, 13.1% were classified into quintile 1. Between the individuals who reported being on a diet or not, there was no difference in the proportions in the quintile 1 (18.2% for both). Of the individuals who reported using supplements, 14.9% were in quintile 1 and among those who did not, 18.9% were in quintile 1. Among those who reported only at-home food consumption, 20.0% were classified into the quintile 1, while among the individuals who reported away-from-home eating, this proportion was 16.3%. Comparing individuals who did not snack and those snacking at least once a day, the proportion of individuals in quintile 1 was 19.9% and 18.0% (Table 3).

## 4. Discussion

A dietary pattern characterized by low consumption of staple and fresh foods and high intake of foods rich in sugars and fats was identified in Brazilian adults and named the energy-dense and low-fiber (ED-LF) dietary pattern.

Greater adherence to the ED-LF pattern, meaning a higher proportion of individuals in the fifth quintile and lower proportion in the first quintile when compared to their counterparts, were observed for the age range over 40 years old, in urban areas, in the highest income category, and for those who reported away-from-home food consumption. Furthermore, individuals who reported not being on a diet and consuming ≥1 snack per day also presented greater adherence to the ED-LF pattern, as the proportion in the quintile 5 was higher than that observed for their counterparts. On the other hand, individuals aged 20 to 40 years old, in rural areas, in the lowest income class, and those who reported at-home only food consumption were less adherent to the ED-LF dietary pattern. As adherence to this dietary pattern increased, the mean intake of sauces, fast foods, and sugar-sweetened beverages increased by 19, 8, and 6 times, respectively, and the mean intake of beans and fruit was reduced by 5 and 4 times, respectively.

Hybrid methods were applied in other studies to identify dietary patterns using dietary components associated with excess weight and extracted similar dietary patterns to that identified in this study [11,12,21,22]. A study examined 4,908 Australian adults and applied Reduced Rank Regression (RRR) using energy density, fiber density, and total sugar as the response variables; they extracted a dietary pattern characterized by a low fiber density and high added-sugar content, which was associated with a greater prevalence of overweight and obesity (Prevalence Ratio = 1.09) [21]. In a Canadian study with 11,748 adults, the PLS method was applied to derive dietary patterns, and energy density, fiber density, and total fat were selected as the response variables; the results showed that the adherence to an “energy-dense, high-fat, and low-fiber density” dietary pattern, characterized by high intake of fast foods, soda, whole grains, solid fats, processed meats, cheeses, baked goods, sugars, and low intake of fruits, vegetables, and yogurt, was associated with increased obesity odds (Odds Ratio = 2.69) [11]. In another Canadian population-based study with 12,049 adults, Ng, Jessri, and L’Abbe [12] also identified a “energy-dense, high-fat, and low-fiber” dietary pattern using energy density, fiber density, and total fat as the response variables. This dietary pattern was characterized by high intake of fast foods, sugar-sweetened beverages, and salty snacks, and low intake of fruits, vegetables, vegetable juices, whole grains, legumes and soy, pasta, and rice dishes, which was associated with increased odds of obesity (OR = 2.40). Livingstone et al. [22] evaluated 625 Australian adults (between 18 and 30 years old) and used RRR to extract dietary patterns using energy density, free sugars, saturated fat, and fiber as the response variables. The first identified dietary pattern was characterized by a high intake of sugar-sweetened beverages, baked goods, and savory products, and low intake of vegetables and pome/berry/stone fruits and was associated with increased odds of overweight/obesity (Odds Ratio = 1.22).

The ED-LF eating pattern contradicts the recommendations of the Brazilian dietary guidelines [23,24] since it is characterized by low consumption of nutritious staples like rice and beans and fresh foods, combined with high consumption of energy-dense, high-sugar, and ultra-processed products. This dietary pattern may increase the risk of nutritional deficiencies and adverse health effects and expresses the changes observed in the Brazilian nutrition scenario, since the regular consumption of beans has been reduced [25,26]. The rice-and-beans combination has a low-energy density, low glycemic index, and high fiber content; therefore, it provides benefits for the prevention of weight gain and non-communicable diseases [27,28,29]. The low consumption of fresh foods, such as vegetables and fruits, is also in line with the trends observed in the Brazilian population, especially because the consumption of fruits and vegetables is restricted by the limited affordability and accessibility related to these items [30,31]. The World Health Organization [18] recommends the consumption of at least 400 g per day of vegetables and fruits; however, the overall mean intake of fruits and vegetables (113.6 g/day) as well as the proportion of those who adhere least to the ED-LF dietary pattern (quintile 1: 189.2 g/day) are far below the recommended level. The positive correlation between bean intake and dietary fiber density probably indicates that beans are an important source of fiber in the Brazilian diet. The ED-LF dietary pattern was also marked by a low intake of water. Encouraging water consumption to replace sugar-sweetened beverages has been adopted as a strategy to reduce weight gain [32,33].

The ED-LF dietary pattern was positively loaded with foods with a high energy density, rich in sugars and fats, and with a low nutrient content, such as sugar-sweetened beverages, fast foods, and sauces that are mostly ultra-processed products. The consumption of these items has increased in recent decades and is associated with weight gain in adults [34,35,36,37,38], and they are usually eaten to replace more complex meals at lunch or dinner [39].

Divergent to our results, in general, adults over 40 years old adopt healthier diets than younger ones [40,41]. The greater adherence to the ED-LF dietary pattern in urban areas is consistent with the unfavorable food environment observed in most medium and big cities, in which the availability and accessibility to low-quality food items, such as ultra-processed foods, is widespread [42,43,44].

Consistently with other studies, no dieters adhered more to the ED-LF pattern, since dieters are usually more careful in making healthier food choices [45,46]. Furthermore, eating-away-from-home and snacking have been associated with a poorer diet characterized by a higher content of calories, added sugars, and solid fats, and reduced intake of whole grains and vegetables [47,48]. Bezerra et al. [49], analyzing data from the same NDS, observed that the most outstanding caloric contribution of away-from-home foods came from alcoholic beverages, snacks, soda, pizza, candies, and sandwiches.

In Brazil, similar dietary patterns have been identified and associated with weight gain indicators in adults. Cunha et al. [10], in a cross-sectional study, investigated 1009 Brazilian adults and identified a direct association between a Western dietary pattern (composed of fast foods, sugar-sweetened beverages, fruit juice, milk and dairy products, cakes, and cookies) and BMI (β = 0.74, *p* = 0.02) and waist circumference among women (β = 13.61, *p* = 0.02). Another cross-sectional study analyzed data from 2034 adults included in the Ribeirão Preto (State of São Paulo) cohort and observed a direct association between a Bar dietary pattern (composed of alcoholic beverages, snacks, pork, sausage, eggs, bacon, seafood, and mayonnaise) and excess weight (BMI ≥ 25 kg/m^2^) and elevated waist circumference in both sexes (Prevalence Ratio = 1.46 and 2.19, respectively) [13].

The identified dietary pattern agrees with the shift in culinary traditions of home-prepared meals to ready-to-eat processed items [50] that are energy-dense, rich in sugars and fats. Besides their deleterious effect on health, such eating habits have negative impacts on environmental sustainability, mainly because the basis of these items are products from agribusiness, which is related to deforestation, reductions in biodiversity, widespread use of pesticides, exhaustion of water reserves, emission of greenhouse gases, intensive use and improper disposal of packaging, and rural exodus, and, finally, its commercialization occurs mainly through unfair commercial practices. Therefore, in addition to the environmental impact, all these conditions have social and economic negative repercussions [51,52,53]. Therefore, healthier and sustainable diets have been encouraged in dietary guidelines, not only to reduce obesity and communicable diseases, but also to minimize the environmental impact of food systems and provide food security. Such diets are mostly plant-based, based on pulses, fruits, vegetables, and nuts, and combined with reduced portions of animal-based foods [24,54,55,56].

The identification of dietary pattern based on the first day of 24hR may be a limitation of the study; however, statistical strategies used to estimate usual intake from two 24hR are limited to calculating individual estimates of food and nutrient intake [57], which are necessary to extract dietary patterns. Additionally, as mentioned by Rodrigues et al. [58], the method applied to obtaining food consumption data in the 2017–2018 National Dietary Survey was subjected to a validation study in which 95 adult and elderly individuals were evaluated. The protein, sodium, and potassium intake estimated from the 24hR was compared with urinary biomarkers, with estimated correlation coefficients of 0.58, 0.31 and 0.30, respectively. Underreporting was observed for protein in 7% of the participants, while 35% underestimated sodium intake, and 20% underestimated potassium intake; these results are comparable with those observed in similar studies [59,60]. Beyond the nationally representative sample, one strength of the study is the use of the 24hR and incorporating procedures to improve data quality, a recommended method for obtaining food consumption data as it enables detailed information and is less subjected to systematic error [61,62,63]. Moreover, adopting a hybrid method and using variables from the obesity causal pathway are adequate strategies to identify dietary patterns [9].

Further nationally representative population-based studies to explore the association between dietary patterns, obesity and metabolic disorders implicated in chronic non-communicable diseases in more depth should incorporate outcome measures related to these conditions in addition to information on dietary expositions.

## 5. Conclusions

The findings emphasize the importance of monitoring the intake of foods that may indicate the healthiness potential of the diet in the nutrition surveillance system [5,64,65], such as staple foods and ultra-processed items, specifically, sugar-sweetened beverages, processed meats, fruits, vegetables, rice, and beans.

The results endorse the adequacy of public health policies, such as adopting a food labeling policy that makes it easier to identify products with higher contents of added sugar, saturated fats, and sodium [66]. Another important measure was the modification of the basic food basket to adapt to the Brazilian dietary guidelines in order to prioritize staple, regional, and fresh foods and to prohibit the inclusion of ultra-processed foods [67]; the products in the official food basket are subject to tax incentives to reduce their price.

In general, an energy-dense and low-fiber dietary pattern, characterized by a low intake of fruits, vegetables, and staple foods and a high intake of fast foods and sugar-sweetened beverages, may contribute to the obesity scenario in Brazil. Individuals over 40 years old, living in urban areas, with a monthly per capita income >2 minimum wage equivalents, and those reporting away-from-home eating have greater adherence to this dietary pattern. The results are relevant to targeting and tailoring strategies of healthy eating promotion to prevent excessive weight gain among adults and guarantee the human right to healthy food.

## Figures and Tables

**Table 1:** Pearson correlation coefficients between predictor loadings, response variables, and scores of the ^1^ PLS-derived energy-dense and low-fiber (ED-LF) dietary pattern in Brazilian adults (20–59 years old). Data from National Dietary Survey, 2017–2018.

	Response Variables			ED-LF Dietary Pattern Score
	Energy Density	Fiber Density	% Energy from Total Fat	PLS
Predictor variable ^†^				
Positive associations				
Solid fats	0.222	−0.077	0.120	0.290
Breads	0.249	−0.094	0.008 ^§^	0.294
Sugar-sweetened beverages	0.136	−0.155	−0.033	0.274
Fast foods	0.243	−0.172	0.064	0.361
Sauces	0.104	−0.086	0.072	0.268
Cheeses	0.081	−0.105	0.144	0.189
Inverse associations				
Rice	−0.204	0.241	−0.113	−0.431
Beans	−0.327	0.673	−0.080	−0.639
Vegetables	−0.226	0.105	0.226	−0.185
Water	−0.072	0.062	−0.005 ^¥^	−0.177
Fruits	−0.269	0.088	−0.089	−0.216
Response variables				
Energy density	1	−0.608	0.299	0.686
Fiber density		1	−0.244	−0.699
% Energy from total fat			1	0.156

^1^ PLS, partial least squares regression. ^†^ Food groups retained in the ED-LF dietary pattern with factor loadings ≥|0.15|. *p*-value for all correlation coefficients was < 0.01 except for ^§^ breads (*p* = 0.178) and ^¥^ water (*p* = 0.455) vs. % energy from total fat.

**Table 2:** Means and 95% confidence intervals of the energy-dense and low-fiber (ED-LF) dietary pattern scores, response variables, and positively and inversely loaded food groups’ intake across the quintiles of the dietary pattern scores in Brazilian adults (20–59 yo). Data from National Dietary Survey, 2017–2018.

	All	Quintiles of the ED-LF Dietary Pattern Factor Scores				
		Quintile 1	Quintile 2	Quintile 3	Quintile 4	Quintile 5
		Means (95% Confidence Intervals)				
Dietary pattern score	0.10 (0.07; 0.13)	−1.53 (−1.57; −1.49)	−0.47 (−0.48; −0.45)	0.04 (0.02; 0.05)	0.53 (0.51; 0.54)	1.54 (1.51; 1.57)
Response variables						
Energy density (kcal/g)	1.78 (1.77; 1.79)	1.35 (1.34; 1.36)	1.52 (1.51; 1.53)	1.71 (1.70; 1.72)	1.93 (1.92; 1.95)	2.27 (2.25; 2.29)
Fiber density (g/1000 kcal)	13.2 (13.0; 13.4)	22.0 (21.7; 22.3)	16.0 (15.8; 16.2)	12.5 (12.3; 12.7)	9.7 (9.5; 9.8)	7.5 (7.4; 7.7)
Total fat intake (%)	29.8 (29.6; 30.0)	27.7 (27.4; 28.1)	29.3 (29.0; 29.7)	30.2 (29.8; 30.6)	30.4 (30.0; 30.7)	30.9 (30.5; 31.3)
Positively loaded food groups (g/day) ^†^	Grams per day					
Solid fats	7.7 (7.4; 8.1)	3.8 (3.4; 4.2)	4.7 (4.3; 5.0)	5.9 (5.5; 6.4)	8.2 (7.7; 8.8)	14.6 (13.7; 15.5)
Breads	50.2 (48.8; 51.6)	27.7 (25.7; 29.6)	35.4 (33.2; 37.6)	42.1 (39.7; 44.5)	55.7 (52.8; 58.6)	82.7 (78.8; 86.6)
Sugar-sweetened beverages	88.7 (83.5; 93.9)	34.7 (27.6; 41.8)	42.2 (35.9; 48.4)	46.1 (40.8; 51.5)	93.0 (83.4; 102.7)	203.5 (188.3; 218.7)
Fast foods	40.3 (38.1; 42.5)	12.5 (10.1; 15.0)	16.6 (14.5; 18.7)	23.6 (21.1; 26.1)	39.5 (35.9; 43.2)	97.3 (90.3; 104.4)
Sauces	1.7 (1.5; 1.9)	0.3 (0.1; 0.4)	0.3 (0.2; 0.5)	0.5 (0.4; 0.7)	0.8 (0.7; 1.0)	5.7 (4.8; 6.6)
Cheeses	6.1 (5.6; 6.7)	2.3 (1.8; 2.8)	2.9 (2.5; 3.4)	4.2 (3.6; 4.9)	6.5 (5.4; 7.5)	13.2 (11.4; 15.0)
Inversely loaded food groups (g/day) ^†^						
Rice	141.1 (137.8; 144.5)	244.8 (235.8; 253.8)	149.0 (143.3; 154.7)	126.2 (120.9; 131.4)	114.1 (109.4; 118.8)	88.5 (84.2; 92.8)
Beans	188.1 (183.3; 192.9)	415.1 (403.8; 426.4)	220.1 (213.5; 226.6)	152.4 (146.3; 158.5)	113.6 (107.9; 119.3)	76.3 (71.6; 81.1)
Vegetables	48.1 (46.2; 50.0)	75.5 (70.1; 80.9)	57.0 (53.2; 60.8)	47.8 (43.6; 52.0)	38.1 (35.4; 40.8)	27.8 (25.6; 30.0)
Water	1231.8 (1205.4; 1258.2)	1530.2 (1467.0; 1591.4)	1334.8 (1279.9; 1389.7)	1208.4 (1159.5; 1257.4)	1124.4 (1082.5; 1166.3)	1022.5 (980.4; 1064.6)
Fruits	65.5 (62.5; 68.5)	113.7 (104.3; 123.0)	83.4 (77.0; 89.8)	61.8 (56.7; 68.9)	47.8 (43.4; 52.2)	30.9 (27.6; 34.2)

^†^ Food groups retained in the ED-LF dietary pattern with a factor loading ≥ |0.15|.

**Table 3:** Distribution (%; 95% confidence interval) of Brazilian adults (20–59 years old) according to sociodemographic variables and quintiles of energy-dense and low-fiber (ED-LF) dietary pattern scores. Data from National Dietary Survey, 2017–2018.

Characteristic	All	Quintiles of the ED-LF Dietary Pattern Factor Scores				
		Quintile 1	Quintile 2	Quintile 3	Quintile 4	Quintile 5
		% (95% Confidence Interval)				
Sex						
Female	50.2	14.1 (13.3; 15.0)	20.2 (19.2; 21.2)	21.9 (20.8; 22.9)	23.0 (21.9; 24.2)	20.8 (19.6; 21.9)
Male	49.8	22.4 (21.3; 23.5)	18.3 (17.2; 19.4)	17.0 (16.0; 18.1)	17.3 (16.3; 18.4)	25.0 (23.7; 26.3)
Age (years old)						
20–40	54.6	19.6 (18.5; 20.7)	21.2 (20.1; 22.3)	19.7 (18.6; 20.8)	19.8 (18.7; 20.9)	19.7 (18.5; 21.0)
>40–59	45.4	17.1 (16.1; 18.1)	17.6 (16.6; 18.7)	19.3 (18.1; 20.4)	20.5 (19.4; 21.6)	25.5 (24.1; 26.9)
Area of residency						
Urban	86.3	16.2 (15.3; 17.1)	18.7 (17.8; 19.6)	19.8 (18.9; 20.7)	20.9 (20.0; 21.8)	24.4 (23.3; 25.6)
Rural	13.7	31.1 (28.9; 33.2)	22.7 (21.2; 24.2)	17.5 (15.9; 19.1)	15.7 (14.2; 17.1)	13.1 (11.6; 14.7)
Monthly per capita family income ^†^						
<0.5	16.1	23.6 (21.7; 25.5)	22.5 (20.7; 24.2)	20.3 (18.5; 22.1)	18.7 (17.1; 20.4)	14.9 (13.2; 16.6)
0.5–1.0	24.4	21.5 (20.0; 23.0)	19.8 (18.5; 21.2)	19.4 (18.0; 20.8)	20.3 (18.9; 21.7)	19.0 (17.3; 20.6)
1.0–2.0	31.3	17.5 (16.2; 18.9)	19.4 (18.1; 20.8)	18.8 (17.6; 20.1)	18.8 (17.5; 20.2)	25.4 (23.3; 27.4)
>2.0	28.2	13.1 (11.8; 14.4)	16.7 (15.1; 18.3)	19.8 (17.9; 21.7)	22.5 (20.8; 24.1)	28.0 (25.9; 30.0)
On a diet						
Yes	13.1	18.2 (16.6; 19.9)	23.6 (21.7; 25.5)	23.6 (21.8; 25.5)	20.1 (18.1; 22.1)	14.4 (12.7; 16.1)
No	86.9	18.2 (17.4; 19.1)	18.6 (17.8; 19.4)	18.8 (17.9; 19.7)	20.2 (19.3; 21.1)	24.1 (23.0; 25.3)
Away-from-home food consumption						
Yes	47.9	16.3 (15.3; 17.4)	17.5 (16.4; 18.6)	19.1 (18.1; 20.2)	20.8 (19.7; 22.0)	26.2 (24.9; 27.5)
No	52.1	20.0 (18.9; 21.1)	20.9 (19.8; 22.0)	19.7 (18.5; 21.0)	19.6 (18.5; 20.7)	19.8 (18.4; 21.2)
Snacking habits						
At least one snack per day	85.7	18.0 (17.1; 18.8)	19.0 (18.2; 19.8)	19.2 (18.4; 20.1)	20.3 (19.4; 21.1)	23.5 (22.5; 24.6)
No snacks	14.3	19.9 (17.6; 22.1)	20.8 (18.4; 23.2)	20.8 (18.7; 23.0)	19.7 (17.4; 21.9)	18.8 (15.9; 21.7)

^†^ Monthly per capita family income categorized in multiples of the country’s official minimum wage in the middle of the surveys [15].

## Data Availability

Publicly available datasets were analyzed in this study. These data can be accessed in: https://www.ibge.gov.br/estatisticas/sociais/populacao/24786-pesquisa-de-orcamentos-familiares-2.html?=&t=microdados, accessed on 30 July 2024.

## Supplementary Materials

The following supporting information can be downloaded at: https://www.mdpi.com/article/10.3390/ijerph21081038/s1, Table S1: Food groups mentioned in the 24 h recall from Brazilian adults. Data from National Dietary Survey, 2017–2018.
