# Supplementary material for: Energy-Dense and Low-Fiber Dietary Pattern May Be a Key Contributor to the Rising Obesity Rates in Brazil
Source: Int J Environ Res Public Health. 2024 Aug 7;21(8):1038. doi: 10.3390/ijerph21081038 (PMC11354081; doi:10.3390/ijerph21081038)
Supplement: Supplementary file 1 [file ijerph-21-01038-s001.zip › ijerph-3091635-supplementary.pdf]

Supplemental Table S1. Food groups mentioned in the 24-hour recall by adults. Brazil, National Dietary Survey, 2017-2018.

Food groups	Food items included in the group	Proportion of report (%)
**Coffee and Tea**	Coffee, latte, cappuccino, and tea.	84.0
**Water**	Water and flavored water.	83.1
**Rice**	Rice and rice dishes.	77.9
**Beans**	Beans, bean dishes, other legumes, meat substitutes.	75.5
**Sugars**	Table sugar, brown sugar, honey, molasses.	66.6
**Beef and Pork**	Beef, beef dishes, preserved meats, viscera, other meats; pork and pork dishes.	52.4
**Bread**	Breads.	51.2
**Vegetables**	Lettuce, kale, cabbage, raw salad, other leafy vegetables, pumpkin, chayote, cucumber, tomato, other non-leafy vegetables, spices.	46.7
**Solid Fats**	Butter, margarine, coconut oil, bacon, whipping cream, sour cream.	38.3
**Poultry**	Poultry and poultry dishes.	35.1
**Fruit Juice**	Fruit juices.	33.5
**Roots and Tubers**	Carrot, sweet potato, potato, cassava, cassava flour, other roots, and tubers.	31.1
**Fruits**	Pineapple, *açaí*, banana, orange, apple, papaya, mango, watermelon, mandarine, grape, dry fruits, other fruits.	28.8
**Fast Food**	Pizzas, French fries, fried and baked snacks, sandwiches, savory pies; savory snacks and chips.	26.5
**Cookies and Crackers**	Cookies and crackers.	24.4
**Pasta**	Pasta, noodles, instant noodles.	21.6
**Sugar-sweetened Beverages**	Sodas, fruit-based processed drinks, diet and light soft drinks, other non-alcoholic beverages.	20.4
**Vegetable oils**	Soy oil, corn oil, olive oil.	15.5
**Eggs**	Eggs and egg-dishes.	14.7
**Cakes and Baked Goods**	Cakes, pies, brownies, donuts, sweet rolls, panettone.	13.7
**Candies, sweets and desserts**	Chocolate, peanut-based sweets, candies, diet and light candies; milk-based sweets, fruit-based sweets, ice cream and popsicle, desserts.	12.3
**Processed Meats**	Processed meats, sausages, bologna, ham, other cold cuts and sausages.	11.9
**Cheeses**	Cheeses, mozzarella, ricotta, grated cheese, and cheese-based dishes.	10.9
**Corn and corn-dishes**	Corn and corn-dishes.	10.0
**Milk**	Whole and skimmed milk and milk-based dishes.	9.6
**Whole grains**	Whole rice, whole noodles, whole crackers and cookies, whole bread, other cereal fibers.	8.9
**Dairy Beverages**	Yogurt, kefir, curd, fermented milk, chocolate milk, dairy, soy milk.	8.7
**Fish**	Fresh fish, canned fish, salted fish, fish dishes, other fish.	8.2
**Non-Caloric Sweeteners**	Non-caloric sweeteners.	7.4
**Soups and broths**	Broth, chowders, and soups.	7.1
**Sauces**	Mayonnaise, mustard, ketchup, shoyu.	5.8
**Alcoholic Beverages**	Spirits, beer, wine.	4.8

Food groups with consumption report <2% were excluded: Nuts, breakfast cereals and supplement
